# Cancer disparities in lean vs. non-lean MASH: insight from a national inpatient sample

**DOI:** 10.1186/s12876-025-04187-1

**Published:** 2025-09-26

**Authors:** Chukwunonso Ezeani, Chidiebele Omaliko, Petr Protiva, Yazan A. Al-Ajlouni, Gyanprakash Ketwaroo, Basile Njei

**Affiliations:** 1 Department of Internal medicine, Baton Rouge General Medical Center, Baton Rouge, LA 70809 USA; 2 Department of Internal Medicine, Brookdale University Hospital, Brooklyn, NY 11212 USA; 3 Division of Digestive Diseases, International Medicine Program (Section of Digestive Diseases), Yale School of Medicine, Yale University, New Haven, CT 06510 USA; 4 Department of Rehabilitation, Montefiore Hospital, Bronx, NY 10466 USA

**Keywords:** Metabolic-dysfunction Associated Steatohepatitis, Metabolic-dysfunction Associated Steatotic Liver Disease, Cancer, Mortality

## Abstract

**Objective:**

To investigate cancer disparities between lean (BMI < 25 kg/m²; < 23 kg/m² for Asians) and non-lean metabolic dysfunction-associated steatohepatitis (MASH) by analyzing the prevalence of the 18 most common cancers in a large U.S. cohort.

**Methods:**

This retrospective cohort study utilized the National Inpatient Sample (2016–2020) with weighted data to project findings to the general population. Patients were categorized as lean or non-lean based on BMI during hospitalization, excluding alternative etiologies via validated algorithms. Outcomes included composite cancer prevalence (primary) and individual cancer prevalence (secondary). Multivariable logistic regression was applied to assess differences.

**Results:**

Among 34,955,252 U.S. hospitalizations, 539,275 patients had MASH (mean age: 64 years for lean vs. 58.8 years for non-lean; >60% female). Lean MASH hospitalizations had higher odds of lung (aOR 1.76, CI 1.33–2.10, *P* < 0.001), colon (aOR 1.23, CI 1.02–1.48, *P* = 0.027), kidney (aOR 1.27, CI 1.10–1.44, *P* = 0.001), liver (aOR 1.21, CI 1.12–1.31, *P* < 0.001), and cervical cancers (aOR 3.25, CI 1.07–9.86, *P* = 0.037), as well as non-Hodgkin’s lymphoma (aOR 1.28, CI 1.08–1.51, *P* = 0.004), but lower odds of endometrial cancer (aOR 0.35, CI 0.25–0.50, *P* < 0.001).

**Conclusion:**

Lean MASH hospitalizations are linked to higher odds of several cancers despite lower BMI, underscoring the need for a nuanced understanding of cancer risks in MASH. BMI alone may not fully capture oncologic risk in this population.

**Supplementary Information:**

The online version contains supplementary material available at 10.1186/s12876-025-04187-1.

## Key messages

What is already known on this topic:

- Metabolic dysfunction-associated steatohepatitis (MASH) is a known risk factor for liver and extrahepatic cancers, predominantly among non-lean individuals. Lean MASH, a metabolic condition without obesity, presents a paradox with unclear cancer risk profiles that have been minimally explored in Western populations.

What this study adds:

- This study identifies a significantly higher prevalence of cancers—including lung, colon, kidney, liver, cervical, and non-Hodgkin’s lymphoma—among lean MASH patients compared to non-lean counterparts. It reveals that body composition may affect specific cancer risks in MASH patients independently of obesity.

How this study might affect research, practice or policy:

- Findings suggest that BMI alone may not fully capture cancer risks in MASH, indicating the need for tailored cancer screenings that incorporate metabolic health in addition to body composition. This approach could inform clinical guidelines, public health initiatives, and future research on cancer prevention in metabolic liver diseases.

## Introduction

Metabolic dysfunction-associated steatotic liver disease (MASLD), previously named Non-alcoholic fatty liver disease (NAFLD), is an umbrella term for conditions ranging from steatosis to Metabolic-dysfunction associated Steatohepatitis (MASH), formerly non-alcoholic steatohepatitis (NASH), with or without fibrosis. These terms were recently proposed by multinational liver societies to improve clarity, reduce stigma, and assist in better patient identification and awareness [1]. MASLD has gained increased significance in the last few decades as they have become a leading cause of chronic liver disease and cirrhosis [2–4]. A significant number of these conditions progress to hepatocellular carcinoma (HCC). Apart from genetic polymorphisms like PNPLA3, it is known that MASH is an important risk factor for HCC [5]. Interestingly, a study found that diabetes, one of the metabolic conditions associated with MASLD, is an independent risk factor for HCC in patients with MASLD/MASH [6]. In addition, another study revealed that increased metabolic factors, including dyslipidemia, obesity, hypertension, and diabetes are associated with higher risk of HCC [7]. The increased risk of cancer in obese patients has been linked to increased tumor-promoting cytokines IL-6 and TNF [8]. Lean MASH thus presents an intriguing paradox in oncology given already established increased risk of cancers, in endometrial cancer for instance. However, most previous studies, as highlighted above emanated mainly from Asia and thus lack global validation.

Observational studies have shown that patients with MASLD are more likely to die from other cancers and cardiovascular risk, compared to hepatocellular carcinoma [9, 10]. There is thus a need to further evaluate factors associated with cancer-related deaths in these patients. Since MASH represents an even higher histopathologic dysfunction in MASLD [11], we hypothesize that disease related dysfunction will be more common in these groups. Obesity represents an important component of metabolic syndrome implicated in MASH/MASLD and a known risk factor for some cancers; however, our anecdotal practice has shown more hospitalizations and deaths in the non-obese arm. However, specific cancer risks in patients with MASH are not known.

Acknowledging a clear gap in detailed risk profiles associated with MASH in different body compositions, this study aims to fill this void by evaluating the prevalence of the most common malignancies across lean and non-lean MASH patients. Utilizing the National Inpatient Sample (NIS) database, a part of the Healthcare Cost and Utilization Project (HCUP) managed by the Agency for Healthcare Research and Quality (AHRQ), we conducted a retrospective cohort analysis. The NIS is the largest publicly available all-payer inpatient care database in the United States, providing a robust framework for tracking a wide array of healthcare outcomes across diverse populations [12]. This database’s extensive reach and detailed diagnostic coding allow for an expansive, representative sample, making it an ideal source for investigating the complex interactions between metabolic dysfunction and cancer risk in a nationally representative cohort. Through this analysis, we seek to establish a clearer understanding of cancer risks tied to metabolic profiles in MASH, with the goal of enhancing targeted screening and refining patient education and counseling strategies.

## Methods

### Study design

We conducted a retrospective cohort study of hospitalizations involving patients with metabolic dysfunction-associated steatotic liver disease (MASLD) using Agency for Healthcare Research and Quality (AHRQ)’s National Inpatient Sample (NIS). AHRQ is a federal agency charged with research in United States health delivery system. The National inpatient sample is a Healthcare Cost and Utilization Project (HCUP) database from AHRQ [13, 14]. It is the largest widely available inpatient database in the United States used to track healthcare utilization, access, charges, quality, and outcomes. The American Hospital Association tracks all hospitalizations in the US annually using regional survey, and stratified by hospital region, bed size, teaching status, urban and/or rural location, and geographical area. A random sample of 20% of are then collected and stored in NIS. The samples were subsequently weighted to make it nationally representative of the United States. Diagnosis is established here using International Classification of Diseases, 10th revision, Clinical Modification (ICD-10-CM) codes, which has been used in NIS since 2015.

### Data collection

Using a validated algorithm, we identified all hospitalizations who were admitted with MASH as primary or secondary diagnosis. We then divided these hospitalizations into those with BMI less than BMI < 25 kg/m^2^, or < 23 kg/m^2^ for Asians vs. those with BMI greater than BMI > 25 kg/m^2^, or > 23 kg/m^2^ for Asians using International Classification of Diseases (ICD) codes (accessory data). We utilized ICD 10 codes for our NIS dataset 2016–2020. ICD 10 codes replaced ICD-9 codes in 2015. All hospitalizations involving patients less than 18 years of age were excluded as we aimed to look at adults only. Information on patients’ demographics, diagnosis (based on ICD-10 codes), hospital and discharge information are all included in NIS dataset. The dataset has been used previously for liver diseases. Missing data was accounted for by removing it from the analysis and not adding it. Supplementary Table 1 A and 1B outline the full ICD codes utilized in this study.

### Study variables/outcomes

Patients demographics including age, gender, and race were extracted. Comorbidities include diabetes, hypertension, hyperlipidemia, smoking status, sarcopenia. Hospital related variables include hospital location, teaching status, and bed size. hospitalization characteristics including length of stay, total hospitalization charges are provided by NIS for each hospitalization.

Hospital location and teaching status are already defined in the NIS, and we utilized these definitions for our analysis. The median household income categories were determined based on the patient’s ZIP code and reported income in the administrative database, using the definitions provided by AHRQ. Data regarding the top 18 most common malignancies is provided in NIS.

### Outcomes

The primary outcome was prevalence of the overall 18 malignancies in lean compared to non-lean MASH hospitalizations. The secondary outcomes were individual prevalence of all malignancies. Multiple confounders were collected and accounted for during analysis including age, gender, race, diabetes, sarcopenia, hypertension, hyperlipidemia, and hospital factors including location, teaching status, and hospital bedsize as well as Elixhauser comorbidity index.

### Statistical analysis

We performed a descriptive analysis to study demographic variables in cases hospitalized with MASH stratified into lean versus non-lean. Continuous variables such as age were reported as mean. We used the discharge weight files provided by HCUP to evaluate overall hospitalization. Weights, strata, and clusters were employed in the analysis to account for the complex survey design of the NIS. A multivariable logistic regression was used to analyze statistical differences in the categorical outcomes. Cancer prevalence among these hospitalizations is reported in proportions. We performed a multivariable logistic regression analysis to study the differences in prevalence of malignancy and overall malignancy among hospitalizations with MASH versus those without MASH. We adjusted for confounding effect of demographic factors such as age, gender, race, primary payer, insurance status, hospital factors including location, bed size and teaching status. We also adjusted for patients’ comorbidities using Elixhauser comorbidity index which utilizes 31 independent patient co-morbidities identified based on ICD-10 coding system. We ran independent logistic regression of covariates and included only those co-variables with *p*-value less than 0.2 into the multivariable logistic regression. We set the cut-off *p*-value of 0.05 for statistical significance. STATA 17 (College station, Texas) was used for all analysis.

### Ethical consideration

As this database contains publicly available deidentified samples, this study was exempt from the Institutional Review Board at our institution.

## Results

Between 2016 and 2020, a total of 34,955,252 hospitalizations were recorded in the United States. Of these, 539,275 involved adults (≥ 18 years) admitted with either a primary or secondary diagnosis of MASH. The analysis revealed distinct differences in the demographic and clinical profiles of lean versus non-lean MASH hospitalizations, as detailed in Table 1. Among the MASH cohort, 324,330 were classified as lean and 214,945 as non-lean, utilizing extracted NIS discharged weight that were utilized for our analysis. The mean age was 64 years in the lean group compared to 58.8 years in the non-lean group. A majority of the hospitalizations were accounted to females, with 60.6% in the lean group and 63.6% in the non-lean group. The predominant racial group was white, comprising 76.8% of the study population. Clinically, hospitalizations involving non-lean MASH patients exhibited a higher prevalence of comorbid conditions such as hypertension (71.8%), hyperlipidemia (42.7%), and diabetes (64.1%), while the smoking rate was notably lower at 0.5%.

**Table 1:** Demographic and clinical characteristics of lean vs. Non-Lean MASH hospitalized patients

Variable	Lean 324,330 (60%)	Non-Lean 214,945 (40%)	*P* value
Age, mean (years)	64.0	58.9	< 0.001
Gender			< 0.001
Female	60.6% (196,502)	63.6% (136,429)	
Male	39.4% (127,828)	36.4% (78,516)	
Race/Ethnicity			< 0.001
White	76.6% (247,552)	76.8% (165,473)	
Black	4.1% (13,309)	5.1% (10,962)	
Hispanic	13.7% (44,580)	13.4% (28,826)	
Other	5.7% (18,430)	4.6% (9,911)	
Charlson Co-morbidity index			
0	5.78 (18,764)	8.58 (18,438)	
1	9.68 (31,395)	12.21 (26,245)	
2	10.34 (33,536)	11.83 (25,428)	
3	13.39 (43,428)	13.23 (28,437)	
4	16.24 (52,671)	14.31 (30,758)	
5	44.57 (144,554)	39.84 (85,639)	
Charlson Co-morbidity index ≥ 3	74.2% (240,702)	67.3% (144,504)	< 0.001
Comorbidities			
Diabetes Mellitus	62.6% (202,206)	64.1% (137,896)	< 0.001
Hypertension	67.9% (220,640)	71.8% (154,779)	< 0.001
Hyperlipidemia	38.7% (125,670)	42.7% (91,770)	< 0.001
Smoking	0.6% (1,960)	0.5% (1,074(	0.0347
Sarcopenia	0.16% (519)	0.10% (215)	0.0196
Type of Admission			< 0.001
Elective Admission	9.7% (31,474)	16.3% (35,095)	
Weekend Admission	22.1% (71,677)	20.7% (44,494)	
Primary Payer Source			< 0.001
Private insurance	61.1% (198,926)	50.3% (107,602)	
Medicaid	11% (35,577)	14.1% (30,353)	
Medicare	23% (74,743)	30.3% (65,000)	
Other Payment Source	2.5% (8,110)	3.0% (6,449)	
Self-Pay	0.2% (649)	0.2% (430)	
No Charge	2.2% (7,144)	2.2% (4,734)	
Median Household income, $			< 0.001
<45 999	29.8% (96,593)	30.0% (64,485)	
46 000–58 999	28.2% (91,710)	29.1% (62,508)	
59 000–78 999	24.5% (79,695)	25.4% (54,698)	
>79 000	17.4% (56,519)	15.6% (33,554)	
Hospital Location Status			< 0.001
Rural	8.1% (26,289)	6.9% (14,856)	
Urban	91.9% (298,041)	93.1% (200,089)	
Hospital Teaching Status			0.041
Teaching	73.9% (239,208)	74.9% (161,964)	
Non-teaching	26.1% (85,122)	25.1% (53,981)	
Hospital Bedsize			< 0.001
Small	17.0% (55,059)	18.4% (39,491)	
Medium	25.4% (82,310)	25.5% (54,814(	
Large	57.6% (186,774)	56.1% (120,688)	

### Composite cancer outcome

Tables 2 and 3 detail cancer outcomes in lean versus non-lean MASH hospitalizations, presenting adjusted odds ratios and *p*-values for individual cancers as well as for a composite of the top 18 malignancies. The analysis highlights a marked disparity in overall cancer prevalence, with lean MASH patients experiencing a composite cancer rate of 8.9%, significantly higher than the 6.5% observed in non-lean patients’ hospitalizations. The adjusted odds ratio of 1.22 (95% CI 1.15–1.29) indicates a statistically significant increased risk of cancer in the lean cohort compared to non-lean MASH patients.

**Table 2:** Cancer outcomes by lean and Non-Lean status among hospitalizations

Outcome	Lean (*N* = 324,330)	Non-Lean (*N* = 214,945)
Breast Cancer	1,946 (0.6%)	860 (0.4%)
Lung Cancer	1,617 (0.5%)	649 (0.3%)
Prostate Cancer	1,063 (0.3%)	215 (0.1%)
Colon Cancer	1,946 (0.6%)	860 (0.4%)
Melanoma	130 (0.04%)	65 (0.03%)
Non-Hodgkin Lymphoma	2,594 (0.8%)	1,075 (0.5%)
Thyroid Cancer	130 (0.04%)	65 (0.03%)
Kidney Cancer	3,632 (1.12%)	1,935 (0.9%)
Pancreatic Cancer	1,294 (0.4%)	645 (0.3%)
Liver Cancer	12,942 (4.0%)	5,612 (2.7%)
Endometrial Cancer	260 (0.08%)	451 (0.21%)
Ovarian Cancer	455 (0.14%)	257 (0.12%)
Esophageal Cancer	195 (0.06%)	107 (0.05%)
Brain Cancer	241 (0.074%)	140 (0.065%)
Gastric Cancer	358 (0.11%)	171 (0.08%)
Cervical Cancer	98 (0.03%)	22 (0.01%)
Rectal Cancer	291 (0.09%)	1,285 (0.6%)
Multiple Myeloma	1,166 (0.36%)	578 (0.27%)
Composite Cancer Outcome	28,848 (8.9%)	13,977 (6.5%)

**Table 3:** Unadjusted and Adjusted Odds Ratios (ORs) of Cancer Outcomes Associated with Lean vs. Non-Lean Status, with Full Multivariate Model CovariatesOdds Ratios for Cancer Outcomes by Lean and Non-Lean Status Among Hospitalizations

Outcome	Unadjusted OR (95% CI)	Adjusted OR (95% CI)^a^	Adjusted *P* value*
Breast Cancer	1.28 (1.07-1.52)	1.18 (0.98-1.42)	0.076
Lung Cancer	2.03 (1.63-2.52)	1.67 (1.34-2.10)	**<0.001**
Prostate Cancer	1.81 (1.35-2.43)	1.18 (0.88-1.60)	0.259
Colon Cancer	1.35 (1.13-1.62)	1.23 (1.03-1.48)	**0.026**
Melanoma	1.19 (0.62-2.29)	1.06 (0.54-2.08)	0.867
Non-Hodgkin Lymphoma	1.43 (1.22-1.68)	1.27 (1.08-1.50)	**0.004**
Thyroid Cancer	1.17 (0.59-2.34)	1.20 (0.60-2.41)	0.596
Kidney Cancer	1.26 (1.11-1.43)	1.26 (1.10-1.44)	**0.001**
Pancreatic Cancer	1.26 (1.01-1.57)	1.09 (0.86-1.37)	0.486
Liver Cancer	1.49 (1.38-1.61)	1.20 (1.12-1.31)	**<0.001**
Endometrial Cancer	0.38 (0.27-0.53)	0.35 (0.25-0.50)	**<0.001**
Ovarian Cancer	1.16 (0.82-1.65)	1.10 (0.77-1.56)	0.611
Esophageal Cancer	1.07 (0.63-1.80)	0.91 (0.53-1.57)	0.748
Brain Cancer	1.14 (0.70-1.84)	1.29 (0.78-2.13)	0.319
Gastric Cancer	1.39 (0.92-2.09)	1.18 (0.79-1.80)	0.432
Cervical Cancer	2.98 (1.01-8.81)	3.25 (1.07-9.88)	**0.037**
Rectal Cancer	1.48 (0.91-2.42)	1.42 (0.87-2.35)	0.161
Multiple Myeloma	1.34 (1.06-1.68)	1.15 (0.91-1.46)	0.227
Composite Cancer Outcome	1.41 (1.34-1.48)	1.22 (1.16-1.29)	**<0.001**

*OR* Odds Ratio, using logistic regression, *CI* Confidence Interval

^a^Adjusted for demographics, hospital characteristics, admission type, year of admission, sociodemographic factors, sarcopenia and all co-morbidities (including the 31 Elixhauser co-morbidities)

*significance if *p *< 0.05

### Common cancer prevalence

**Fig. 1:**
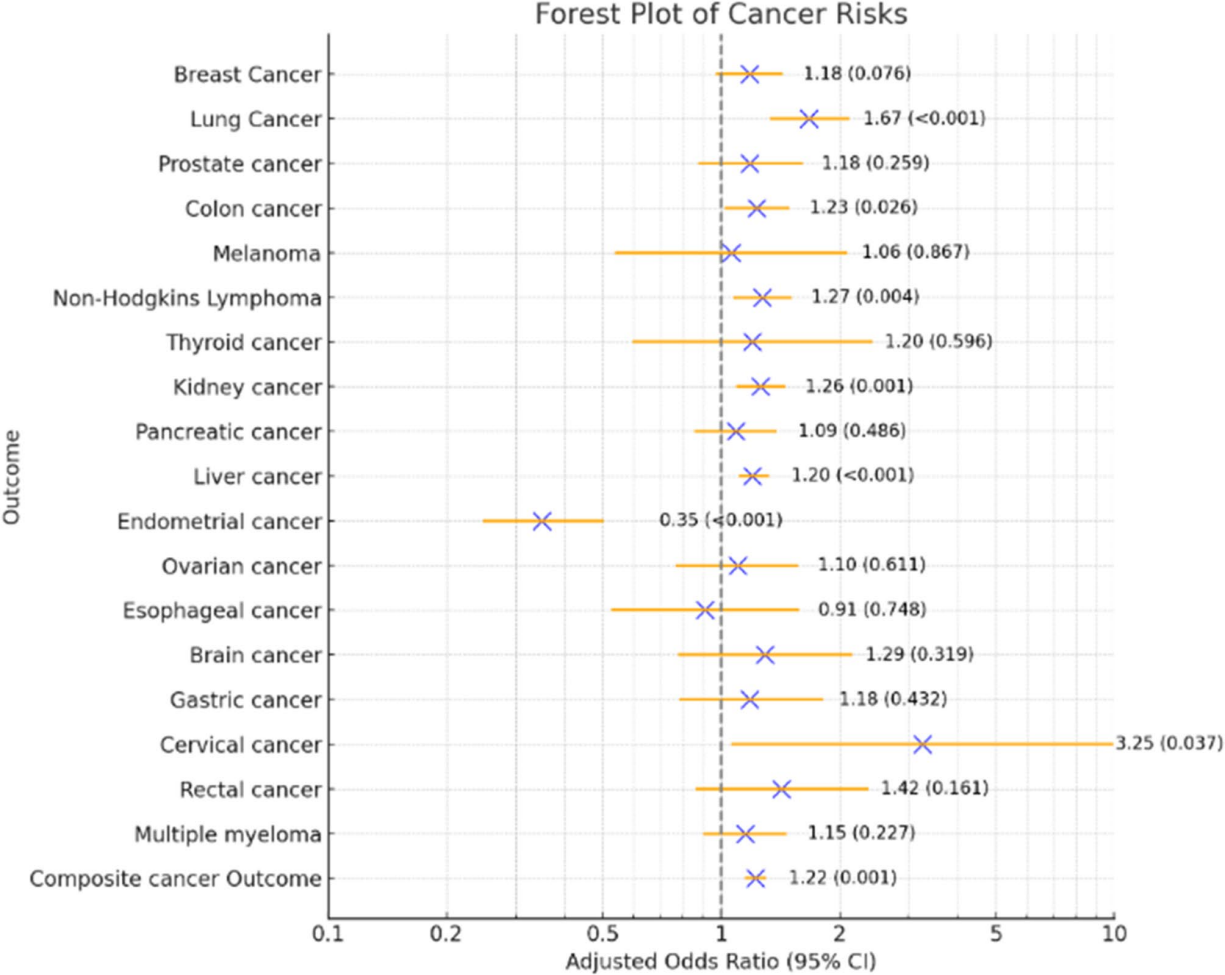
Adjusted Odds Ratios for Various Cancer Outcomes. Legend: This forest plot illustrates the adjusted odds ratios (ORs) for the risk of different types of cancer. Each point represents the OR for a specific cancer type, with horizontal lines indicating the 95% confidence intervals. Values next to each point denote the OR followed by the corresponding *p*-value in brackets, providing a measure of statistical significance. A vertical dashed line at OR = 1 indicates no effect

When examining specific cancer types, lean MASH hospitalizations demonstrated a higher prevalence of several cancers. Notably, the adjusted odds for lung cancer were 1.67 times greater in lean than in non-lean hospitalizations (95% CI 1.34–2.10, *P* < 0.001), highlighting a significant risk differential. Similarly, lean patients’ hospitalizations showed increased prevalences of colon cancer (0.6% vs. 0.4%, aOR 1.23, CI 1.02–1.48), non-Hodgkin’s lymphoma (0.04% vs. 0.03%, aOR 1.28, CI 1.08–1.51), kidney cancer (1.12% vs. 0.9%, aOR 1.27, CI 1.10–1.44), and liver cancer (4.0% vs. 2.7%, aOR 1.21, CI 1.12–1.31). Cervical cancer also showed a markedly higher rate in the lean group (0.03% vs. 0.01%, aOR 3.25, CI 1.07–9.86). Conversely, non-lean hospitalizations were found to have a higher prevalence of endometrial cancer (0.08% vs. 0.21%, aOR 0.35, CI 0.25–0.50), indicating a different cancer risk profile. For other cancers including breast, prostate, melanoma, thyroid, pancreatic, ovarian, esophageal, brain, gastric, rectal cancers, and multiple myeloma, no significant differences in prevalence rates were observed between the lean and non-lean groups, as illustrated in Fig. 1.

In the sex-stratified analysis (Table 4), lean female MASH patients exhibited significantly higher adjusted odds of breast cancer (aOR 115, CI 37.24–360.04, *P* < 0.001) compared to non-lean females. Conversely, lean females had lower odds of kidney cancer (aOR 0.576, CI 0.51–0.65, *P* < 0.001), liver cancer (aOR 0.463, CI 0.43–0.50, *P* < 0.001), and non-Hodgkin’s lymphoma (aOR 0.687, CI 0.59–0.80, *P* < 0.001).

**Table 4:** Sex-Stratified associations between lean status and Site-Specific Cancer outcomes: adjusted ORs and 95% CI

Cancer Outcomes	OR in Female (CI)	*P* value
Breast	115 (37.24–360.04)	0.000
Lung	0.998 (0.82–1.22)	0.986
Prostate	1	--
Colon	0.828 (0.70–0.98)	0.031
Melanoma	0.513 (0.26–0.99)	0.050
Non-Hodgkin’s Lymphoma	0.687 (0.59–0.80)	0.000
Thyroid cancer	1.007 (0.51–1.98)	0.978
Kidney cancer	0.576 (0.51–0.65)	0.000
Pancreatic cancer	0.780 (0.63–0.97)	0.026
Liver cancer	0.463 (0.43–0.50)	0.000
Endometrial	--	--
Ovarian	--	--
Esophageal	0.139 (0.07–0.28)	0.000
Brain	0.569 (0.36–0.91)	0.018
Gastric	0.691 (0.46–1.02)	0.066
Cervical	--	--
Rectal	0.7138 (0.46–1.10)	0.125
Multiple Myeloma	0.579 (0.46–0.72)	0.000
Composite Cancer	0.640 (0.61–0.67)	0.000

*OR* Odds Ratio, using logistic regression, *CI* Confidence Interval

## Discussion

In this extensive population-based study, we systematically assessed the prevalence of the most common cancers among patients hospitalized with MASH using the National Inpatient Sample. To date, *and to the best of our knowledge*, this is the first study to investigate disparities in cancer outcomes among lean and non-lean MASH hospitalized patients. Our findings indicate a notably higher risk of cervical cancer in lean MASH patients, with odds three times greater than those observed in the non-lean cohort. Additionally, the lean group exhibited increased prevalence of non-Hodgkin’s lymphoma, lung, colon, kidney, and liver cancers when compared to their non-lean counterparts. Conversely, among non-lean hospitalizations, a higher incidence of endometrial carcinoma was demonstrated. Notably, the analysis revealed no significant differences in the prevalence of melanoma, multiple myeloma, breast, prostate, thyroid, pancreatic, ovarian, esophageal, brain, gastric, and rectal cancers between the two groups. These findings highlight the complex relationship between body composition, metabolic dysfunction, and cancer risk, highlighting the need for targeted screening and preventive measures based on metabolic health status and body composition in MASH hospitalized patients.

Our study adds to the understanding of increased cancer risk in hospitalized patients with MASLD, including MASH seen in previous studies that revealed increased mortality rate among all stages of steatotic liver disease, including MASH [15–17] by stratifying this risk based on BMI using a nationwide population-based study. The most well described in the literature is the progression of from MASLD through MASH, with or without fibrosis to cirrhosis to hepatocellular carcinoma, and the rate of this complication has been increasing over the years [18]. MASH is an independent risk factor for HCC and accounts for about 7% of HCC globally [2]. Interestingly, death in hospitalized patients with MASLD, inclusive of those with MASH have been noted to occur more often from extrahepatic cancers [2]. 

Our study corroborates existing research linking diabetes and hepatocellular carcinoma (HCC), as well as various extrahepatic solid organ cancers such as prostate, breast, esophagus, colon, lung, pancreas, kidney, and bladder [16]. Notably, while we observed this continued association in cancers like HCC, colon, lung, and kidney, our data also revealed a heightened risk for non-Hodgkin’s lymphoma and cervical cancer within the lean MASH cohort. This expands the conventional understanding that primarily links metabolic dysfunctions such as obesity and diabetes with cancer, suggesting that additional genetic or environmental factors may influence cancer susceptibility in this population.

Previous observational studies have often evaluated cancer risks within the entire spectrum of MASLD but lacked a global perspective. Our findings contribute to this narrative by clarifying that while MASLD is commonly associated with increased risks for hepatocellular and extrahepatic cancers, the specific relationship between MASH, body composition, and cancer risk remains complex. This emphasizes the need for further investigations that are globally representative and stratify patients from initial steatosis through MASH to cirrhosis, thereby enhancing our understanding of how obesity interplays with metabolic liver disease to modulate cancer risk. Additionally, our analysis identified an increased risk of endometrial cancer in the obese MASH cohort, aligning with previous studies that have highlighted the role of hormonal imbalances associated with obesity [19]. This observation reinforces the established link between excess adiposity and hormonal cancers, further emphasizing the importance of managing body weight as part of a comprehensive cancer prevention strategy in patients with MASH. This finding prompts a deeper exploration of hormonal pathways and their modulation by metabolic states in future research, which could unveil new preventive and therapeutic targets. Finally, while our study did not yield statistically significant differences, we observed trends suggesting potentially elevated risks for several cancers, including breast, prostate, thyroid, pancreatic, ovarian, brain, gastric cancers, melanoma, and multiple myeloma. Although these findings did not reach statistical significance, they contribute to the growing body of evidence suggesting a link between excess body fat and the pathogenesis of multiple malignancies. Despite the absence of conclusive significance, these trends emphasize the importance of considering the multifaceted interplay between metabolic dysfunction, adiposity, and cancer susceptibility.

Finally, our sex-stratified analysis revealed striking disparities: lean female MASH patients had 115-fold higher odds of breast cancer but lower odds of liver and kidney cancers compared to non-lean females. While the extreme odds for breast cancer may reflect residual confounding (e.g., undiagnosed genetic risk), the inverse association with hepatocellular and renal cancers aligns with evidence that obesity-driven metabolic dysfunction (e.g., hyperinsulinemia, adipokine imbalance) disproportionately promotes these malignancies in non-lean populations. These contrasts underscore that *lean MASH* may represent a distinct phenotype where cancer risk is mediated by non-adipose mechanisms, such as estrogen signaling in breast tissue or sarcopenia-related metabolic dysregulation. Future studies should explore whether sex hormones or body composition (e.g., visceral fat vs. muscle mass) modulate these risks independently of BMI.

### Clinical implications

The observed disparities in cancer prevalence between hospitalized lean and non-lean MASH patients underscore the critical need for tailored screening protocols that account for metabolic health status and body composition. Clinicians should recognize the heightened cancer risk among hospitalized lean MASH patients and consider incorporating regular cancer screenings into their management plans. Given the threefold higher odds of cervical cancer observed in hospitalized lean MASH patients compared to their non-lean counterparts, routine cervical cancer screenings should be prioritized in this population. Early detection through regular screenings can facilitate timely intervention and improve patient outcomes.

Managing MASH requires a holistic approach that extends beyond liver health to encompass comprehensive care targeting metabolic dysregulation and associated cancer risks. Clinicians should adopt a multifaceted management strategy that addresses both liver-specific concerns and modifiable risk factors implicated in cancer pathogenesis. Obesity and diabetes, known contributors to cancer development, should be actively addressed in the management of MASH patients. Interventions aimed at weight management, lifestyle modifications, and glycemic control are crucial for mitigating cancer risk and improving overall health outcomes.

### Public health implications

Moreover, considering the findings highlighted in this analysis among hospitalized MASH patients, particularly among lean individuals, public health initiatives must prioritize raising awareness of these risks. Health promotion campaigns should be tailored to educate the public, with a specific emphasis on lean individuals, about the importance of maintaining a healthy weight and adopting lifestyle modifications to mitigate cancer risk. By disseminating targeted information through various channels, such as social media, community outreach programs, and healthcare facilities, public health campaigns can empower individuals to make informed decisions regarding their health and well-being. Policymakers play a pivotal role in addressing the public health implications of MASH-associated cancer risks. It is imperative for policymakers to integrate cancer screening and prevention strategies into existing healthcare systems, ensuring equitable access to preventive healthcare services for all individuals, including those diagnosed with MASH. This may entail implementing policies that facilitate early detection and intervention, such as reducing barriers to screening programs and promoting the adoption of evidence-based guidelines for cancer prevention and management. By enacting comprehensive policy interventions, policymakers can contribute to reducing the burden of MASH-associated cancers and improving health outcomes on a population level.

### Limitations

We acknowledge several limitations in this study. First, the diagnosis of MASH is established using ICD-10 codes from a nationwide database, which may introduce selection bias due to potential misclassification or incorrect coding. Secondly, the lack of longitudinal follow-up in our dataset prevents us from determining if some lean patients were initially non-lean and lost weight due to underlying cancer or other health issues. This limitation is inherent to the use of the NIS, which does not track patients over time. This underscores the need for longitudinal studies to confirm our findings and to better understand the temporal dynamics between body weight changes and cancer development. Thirdly, as this is a cross-sectional study, we cannot measure causality, and only associations can be assumed. Additionally, it is important to note that the unit of analysis in this study is hospitalizations rather than individual patients. As a result, patients who are hospitalized more than once may be counted multiple times, which could impact the interpretation of the data. Despite employing multivariable analysis to control for patient demographics and comorbidities, residual confounding by unmeasured or inadequately measured factors cannot be ruled out. Factors such as lifestyle choices, socioeconomic status, and detailed clinical history were not available in the NIS, which could influence both MASH progression and cancer risk. Furthermore, the use of administrative data restricts the ability to explore deeper clinical nuances that might affect disease classification and outcomes. Clinical subtleties such as the severity of steatohepatitis, patient medication histories, and finer details of metabolic health are beyond the scope of what administrative codes can capture. Moreover, it is crucial to specify that this study is based on hospitalization data from the NIS. While this database could be externally validated to US hospitals, caution should be exercised in generalizing the findings to the broader US population. Moreover, we acknowledge that BMI is frequently underreported in administrative data, which may affect the reliability of our findings. Furthermore, BMI coding (e.g., Z68.2) may overlap lean and non-lean ranges, especially across ethnic groups (e.g., Asians), potentially leading to misclassification. This undercoding could potentially lead to misclassification and bias in our results. Additionally, BMI cutoffs may differ in older adults due to age-related changes in body composition; however, the database did not support age-specific BMI categorization. Therefore, caution should be exercised when interpreting the associations between BMI and cancer risk in this study. Additionally, while our dataset includes hospitalizations from 2016 to 2020, the structure of the NIS only allows analysis at the yearly level making it difficult to differentiate between hospitalizations pre- and post-COVID-19 Pandemic in early 2020. Given the potential impact of the pandemic on cancer detection, screening delays, and healthcare access, this represents an important limitation. However, due to the limited post-pandemic observation period and the lack of monthly-level data, stratifying our analysis by pre- and post-pandemic years could introduce bias. Finally, while this study contributes valuable insights into the role of BMI in cancer pathogenesis among MASH patients, these findings need to be interpreted with caution and verified through additional studies that can address these limitations. This study is among the first to elucidate the potential cancer risk disparities within this patient population, laying the groundwork for more detailed investigations in the future.

### Future research

Future research should prioritize validating these findings across diverse datasets and patient populations to ascertain the generalizability and robustness of the observed associations. Studies employing longitudinal databases that track patients over time would be particularly valuable, enabling researchers to observe changes in metabolic status and corresponding shifts in cancer risk profiles. This longitudinal approach would also facilitate a better understanding of the temporal relationship between MASH and cancer development. Prospective studies, ideally with a multi-center design, are crucial to explore not only the cancers examined in this study but also other malignancies that may be associated with MASH. Such studies should incorporate detailed metabolic and genetic profiling to dissect the mechanisms by which metabolic dysfunction influences cancer pathogenesis. Moreover, it would be instructive to explore the impact of interventions targeting metabolic dysfunctions, such as pharmacological treatments, lifestyle modifications, and bariatric surgery, on cancer incidence in MASH patients. This could offer insights into potential preventive strategies that could be implemented in high-risk populations. In addition to clinical studies, bioinformatics approaches involving big data analytics and machine learning could be employed to identify novel biomarkers and potential therapeutic targets for cancers associated with MASH. These computational studies could leverage existing medical datasets to predict cancer risk and outcomes more accurately in patients with various forms of metabolic dysfunction. Ultimately, these efforts should aim to foster a multidisciplinary approach, combining clinical, genetic, and public health strategies to mitigate the elevated cancer risks associated with metabolic dysfunction-associated steatohepatitis.

## Conclusion(s)

In conclusion, this comprehensive population-based study conducted in the United States has elucidated significant associations between body composition and cancer risks in hospitalized patients admitted with MASH. Specifically, hospitalized lean individuals with MASH were found to have an elevated risk of HCC, lung, colon, kidney, and cervical cancers, as well as non-Hodgkin’s lymphoma, as compared to hospitalized non-lean MASH patients. Conversely, those hospitalized with non-lean MASH exhibited a higher risk of endometrial cancer as compared to their lean counterparts. These findings highlight the need for clinicians to consider body composition as a factor in cancer screening and prevention strategies for patients with MASH. While the results are compelling, they necessitate further validation through longitudinal studies to confirm these associations and to explore the underlying mechanisms. This study underscores the importance of tailored approaches in management and monitoring of cancer risks in different MASH patient subgroups based on their metabolic profiles.

## Supplementary Information

Supplementary Material 1.

## Data Availability

Data is available upon reasonable request from the corresponding author.
