# Supplementary material for: Cancer disparities in lean vs. non-lean MASH: insight from a national inpatient sample
Source: BMC Gastroenterol. 2025 Sep 26;25:659. doi: 10.1186/s12876-025-04187-1 (PMC12465481; doi:10.1186/s12876-025-04187-1)
Supplement: Supplementary file 1 — Supplementary Material 1. [file 12876_2025_4187_MOESM1_ESM.docx]

**Supplementary Table 1A.** ICD Classification for MASH and Related Cancer Diagnoses

| Variable | ICD Codes |
| --- | --- |
| MASH | K7581 |
| Non-Lean | Z682; Z683; Z684; E660; E661; E662; E668; E669 |
| Breast Cancer | C50 |
| Lung Cancer | C34 |
| Prostate Cancer | C61 |
| Colorectal Cancer | C18; C19; C20 |
| Bladder Cancer | C67 |
| Melanoma | C43 |
| Non-Hodgkin’s Lymphoma | C82; C83; C84; C85; C96 |
| Thyroid Cancer | C73 |
| Kidney cancer | C64 |
| Leukemia | C91; C92; C93; C94; C95 |
| Pancreatic cancer | C25 |
| Liver Cancer | C22 |
| Endometrial Cencer | C54 |
| Ovarian Cancer | C56 |
| Esophageal cancer | C15 |
| Brain and Nervous system cancer | C70; C71; C72 |
| Gastric Cancer | C16 |
| Cervical Cancer | C53 |
| Rectal Cancer | C20 |
| Multiple myeloma | C90 |

**Supplementary Table 1B.** ICD Classification For Comorbidities Included in Analysis

| Comorbidities | ICD-10 Codes |
| --- | --- |
| Diabetes | E10, E11, E12, E13, E14 |
| Hypertension | I10, I11, I12, I13, I15 |
| Chronic Kidney Disease | N18, |
| COPD | J42, J43, J47 |
| Asthma | J45 |
| Obesity | E66 |
| Hyperlipidemia | E78.1; E78.2; E78.3; E78.4; E78.5 |
| Coronary artery disease | I25.7, I25.11 |
| Prior myocardial infarction | I25.2 |
| Cerebrovascular disease | I60; I61; I62; I63; I65; I66; I67; I68 |
| Stroke | I63 |
| Aortic Stenosis | I35.0; I06.0 |
| Heart Failure | I09.9; I11.0; I13.0; I13.2; I25.5; I42.0; I42.5-I43.9; I43; I50; P29.0 |
| Atrial Fibrillation, Flutter | I48 |
| Cardiogenic shock | R57.0 |
| Sepsis | A40; A41 |
| Acute Kidney Injury | N17 |
| Hemodialysis for ARF | [5A1D70Z](https://www.icd10data.com/ICD10PCS/Codes/5/A/1/D/5A1D70Z); [5A1D80Z](https://www.icd10data.com/ICD10PCS/Codes/5/A/1/D/5A1D80Z); [5A1D90Z](https://www.icd10data.com/ICD10PCS/Codes/5/A/1/D/5A1D90Z) |
| Upper GI bleed | [K20.81](https://www.icd10data.com/ICD10CM/Codes/K00-K95/K20-K31/K20-/K20.81); [K20.91](https://www.icd10data.com/ICD10CM/Codes/K00-K95/K20-K31/K20-/K20.91); [K22.11](https://www.icd10data.com/ICD10CM/Codes/K00-K95/K20-K31/K22-/K22.11); [K25.0](https://www.icd10data.com/ICD10CM/Codes/K00-K95/K20-K31/K25-/K25.0); [K25.2](https://www.icd10data.com/ICD10CM/Codes/K00-K95/K20-K31/K25-/K25.2); [K25.4](https://www.icd10data.com/ICD10CM/Codes/K00-K95/K20-K31/K25-/K25.4); K25.6; K26.0; K26.2; K26.4; K26.6; K27.0; K27.2; K27.6; K28.0; K28.2; K28.4; K28.6; K29.91; K29.21; K29.31; K29.51; K29.61; K29.71; K29.81; K29.91; K31.811; K31.82; |
| Lower GI bleed | K92.1; K92.2; |
| Hypovolemia | E86.0; E86.1; E86.9 |
| Electrolyte imbalance | E22.2; E87 |
| Anemia | D50; D51; D52; D53; D54; D55; D56; D57; D58; D59; D60; D61; D62; D63; D64 |
| Malnutrition | E40; E541; E42; E43; E44; E45; E46 |
| Clostridium difficile | A04.71; A04.72 |
| Bowel obstruction | K56 |
| Chronic steroid use | Z79.5 |
| Pneumonia | J12; J13; J14; J15; J16; J17; J18 |
| Pulmonary embolism | I26; I27.82 |
| Ischemic bowel | K55.0 |
| Sickle cell disease | D57 |
| AIDS | B20-B22; B24 |
